# Cardiovascular–Kidney–Metabolic (CKM) Syndrome as Independent Risk Factor for Pneumococcal Pneumonia: Evidence from a Territory-Wide Study

**DOI:** 10.3390/microorganisms14020439

**Published:** 2026-02-12

**Authors:** Wang Chun Kwok, Isaac Sze Him Leung, Chun Ka Wong, David Chi Leung Lam, Mary Sau Man Ip, Kelvin Kai Wang To, James Chung Man Ho, Desmond Yat Hin Yap

**Affiliations:** 1 Division of Respiratory Medicine, Department of Medicine, The University of Hong Kong, Queen Mary Hospital, Hong Kong, China; kwokwch@hku.hk (W.C.K.); dcllam@hku.hk (D.C.L.L.); msmip@hku.hk (M.S.M.I.); 2 Department of Statistics, The Chinese University of Hong Kong, Hong Kong, China; shleung@cuhk.edu.hk; 3 Division of Cardiology, Department of Medicine, The University of Hong Kong, Queen Mary Hospital, Hong Kong, China; wongeck@hku.hk; 4 Department of Microbiology, School of Clinical Medicine, Li Ka Shing Faculty of Medicine, The University of Hong Kong, Hong Kong, China; kelvinto@hku.hk; 5 Division of Nephrology, Department of Medicine, The University of Hong Kong, Queen Mary Hospital, Hong Kong, China

**Keywords:** cardiovascular–kidney–metabolic syndrome, mortality, pneumococcal pneumonia, severe respiratory failure, acute kidney injury

## Abstract

Cardiovascular–kidney–metabolic (CKM) syndrome is an increasingly recognized condition that highlights the interaction between three important medical co-morbidities. Whether the presence of CKM syndrome may increase the risk of in-hospital adverse outcomes in patients with pneumococcal pneumonia has not been investigated. We conducted a territory-wide retrospective study on adults hospitalized for pneumococcal pneumonia between 1 January 2016 and 31 December 2024 in Hong Kong. In-patient mortality, severe respiratory failure (SRF) and acute kidney injury (AKI) were compared among patients with cardiovascular–kidney–metabolic (CKM) syndrome at different stages. Subgroup analyses were performed in patients who have or have not received a pneumococcal vaccine. In total, 2192 patients were hospitalized for pneumococcal pneumonia in the study period, with 1005 (45.8%), 373 (17.0%), 684 (31.2%) and 130 (5.9%) at stage 0–1, 2–3, 4a and 4b CKM syndrome. A higher stage of CKM syndrome was associated with increased risks of death during index admission, SRF and AKI. The adjusted odds ratios (aOR) for CKM stage 4a and 4b for death during index admission were 1.82 (95% CI 1.25–2.64) and 10.92 (95% CI 6.82–17.49) respectively (*p* = 0.002 and <0.001). The aOR for SRF for CKM stage 2–3, 4a and 4b were 1.43 (95% CI 1.01–2.03), 1.88 (95% CI 1.39–2.54) and 28.42 (95% CI 16.92–47.74) respectively (*p* = 0.042, <0.001 and <0.001). The aOR for AKI for CKM syndrome stage 2–3, 4a and 4b were 2.25 (95% CI 1.53–3.29), 3.00 (95% CI 2.14–4.22) and 4.30 (95% CI 2.69–6.88) (*p* < 0.001 for all). Subgroup analysis showed consistent results among those who have or have not received a pneumococcal vaccine within the 12 months preceding the index admission date. CKM syndrome, especially at a higher stage, constitutes an independent risk factor for severe in-hospital outcomes in adults hospitalized for pneumococcal pneumonia.

## 1. Introduction

*Streptococcus pneumoniae* is a fastidious Gram-positive, alpha-hemolytic bacterium that grows best in 5 to 10% carbon dioxide and requires a source of catalase to grow on agar plates. It is one of the commonest bacteria causing community-acquired pneumonia [1], which is associated with a high mortality rate of up to 30%. In 2019, there were almost 1.4 million deaths attributable to pneumococcal pneumonia (50% affecting patients over 70 years old), and it was the leading cause of deaths from infectious disease worldwide [2]. Advanced age [3], chronic illnesses such as chronic obstructive pulmonary disease (COPD), and immunocompromised state [4] have been reported as risk factors for severe infection, complications, and mortality. Pneumococcal pneumonia is transmitted from person to person by intimate contact or aerosol.

The classical symptoms of pneumococcal pneumonia include acute onset fever, chills, cough, pleuritic chest pain, and rusty-colored sputum. Pneumococcal pneumonia can be complicated by the development of empyema thoracis, bacteremia, meningitis, endocarditis, pericarditis, myocarditis, septic arthritis, and peritonitis [4,5]. Acute cardiac events such as acute myocardial infarction, cardiac arrhythmia and heart failure can also complicate the course of pneumococcal pneumonia [6]. One of the most severe forms of the infection is called invasive pneumococcal disease (IPD), which is defined as an infection in a normally sterile body site, such as blood, cerebrospinal fluid, pleural fluid, synovial fluid, or pericardial fluid. As such, IPD can present as bacteremia, meningitis, spontaneous bacterial peritonitis, septic arthritis, endocarditis, epidural abscess, dental abscess and osteomyelitis.

To protect susceptible subjects from the infection, vaccines have been developed which are proven to be effective at reducing the morbidity and mortality associated with pneumococcal disease. Two types of pneumococcal vaccines are approved and have defined indications for use, including a pneumococcal polysaccharide vaccine and a pneumococcal conjugate vaccine. Despite vaccination, pneumococcal pneumonia remained prevalent and a phenomenon known as serotype replacement emerged, which is the increase in non-vaccine serotypes in a population following a reduction in vaccine-targeted strains [7]. A recently published systematic review reported that the most common serotype in adults was serotype 3 followed by serotypes 19A and 11A, which were included in current vaccines. Emergence of non-vaccine serotypes was also observed in the systemic review [2]. The observation of serotype replacement calls for the continuous advancement of vaccination against *Streptococcus pneumoniae*.

Medical co-morbidities can co-exist and interact with each other and may compound the impact of acute illnesses such as infections. Cardiovascular–kidney–metabolic (CKM) syndrome, proposed by the American Heart Association (AHA), stratifies patients with various cardio–renal–metabolic risk factors or outcomes into a syndrome with four stages based on the presence of risk factors or diseases [8]. CKM syndrome is increasingly recognized as a systemic disorder characterized by pathophysiological interactions among metabolic risk factors, chronic kidney disease (CKD), and the cardiovascular system leading to multiorgan dysfunction and a high rate of adverse cardiovascular outcomes.

The stages of CKM syndrome are defined according to the AHA proposal [8]: stage 0—no CKM health risk factors, which refers to individuals without overweight/obesity, metabolic risk factors (hypertension, hypertriglyceridemia, metabolic syndrome (MetS), diabetes), CKD, or subclinical/clinical cardiovascular disease (CVD); stage 1—excess and/or dysfunctional adiposity, which refers to individuals with overweight/obesity, abdominal obesity, or dysfunctional adipose tissue, without the presence of other metabolic risk factors or CKD; stage 2—metabolic risk factors and CKD, which refers to individuals with metabolic risk factors (hypertriglyceridemia, hypertension, MetS, diabetes) or CKD; stage 3—subclinical CVD in CKM, which includes subclinical atherosclerotic CVD or subclinical heart failure among individuals with excess/dysfunctional adiposity, other metabolic risk factors, or CKD; stage 4—clinical CVD in CKM, which includes clinical CVD (coronary heart disease, heart failure, stroke, peripheral artery disease, atrial fibrillation) among individuals with excess/dysfunctional adiposity, other metabolic risk factors, or CKD. Stage 4 CKM syndrome is further subclassified into Stage 4a, which had no renal failure, and stage 4b, in which renal failure is present.

Previous studies have indicated that higher CKM syndrome stages were associated with an increased risk of all-cause mortality, particularly among younger adults [9]. Other investigators have also reported associations between CKM syndrome and adverse clinical outcomes in patients undergoing non-cardiac surgery, dementia, renal calculi and CKD [10,11,12]. However, being a relatively new clinical entity, evidence on the association between CKM syndrome and the outcome of pneumococcal pneumonia is lacking. To complicate the situation further, CKM syndrome is linked to systemic inflammation and oxidative stress and hence may influence the outcomes of patients suffering from active infections [13,14,15,16]. To resolve these issues, this study aimed to examine the relationship between CKM syndrome at different stages and the occurrence of severe in-hospital outcomes among adults hospitalized for pneumococcal pneumonia. The results from this study will help risk stratify patients with pneumococcal pneumonia and guide clinical decisions on vaccinations.

## 2. Materials and Methods

### 2.1. Study Design and Participants

This territory-wide retrospective study evaluated the relationships between CKM syndrome at different stages and mortality and serious in-hospital outcomes in adult patients hospitalized for pneumococcal pneumonia. Adult patients aged 18 or above who were admitted to public hospitals in Hong Kong for pneumococcal pneumonia between 1 January 2016 and 31 December 2024 were included. Patients were identified from the Clinical Data Analysis and Reporting System (CDARS) of the Hospital Authority (HA). The CDARS is an electronic health record database managed by the HA—a public healthcare service provider that covers over 90% of the Hong Kong population and has done so since 1993 [17,18,19]. The CDARS captures medical information including the diagnosis, drug prescription details, demographic, admission, medical procedures, and laboratory results of the patients who attended public medical services operated by the HA. The diagnosis of pneumococcal pneumonia was identified using the International Classification of Diseases, Ninth Revision (ICD-9) code of 481 (Pneumococcal pneumonia [*Streptococcus pneumoniae* pneumonia]). Patients with an ICD-9 code of 481 as the primary and secondary diagnosis during index admission were identified in the CDARS.

Patients were stratified into the following subgroups based on the stage of CKM syndrome at the time of index admission: stage 0–1, 2–3, 4a and 4b [8].

### 2.2. Outcome Measurements

The main outcomes of interest were: (1) death during index admission; (2) severe respiratory failure (SRF) requiring invasive or non-invasive mechanical ventilation and high-flow nasal cannula; and (3) acute kidney injury (AKI). AKI was defined according to the RIFLE criteria [20].

The RIFLE criteria classified the renal outcomes as follows: Risk (R): Serum creatinine increased by 1.5 times or eGFR decreased by >25%, urine output <0.5 mL/kg/h for 6 h. Injury (I): Serum creatinine increased by 2 times or eGFR decreased by >50%, urine output <0.5 mL/kg/h for 12 h. Failure (F): Serum creatinine increased by 3 times or eGFR decreased by >75%, urine output <0.3 mL/kg/h for 24 h or anuria for 12 h. Loss (L): Persistent AKI, complete loss of kidney function for more than 4 weeks. End-stage (E): End-stage renal disease (need for dialysis for more than 3 months).

### 2.3. Statistical Analysis

Descriptive tables were created to present the incidence rates of severe in-hospital outcomes stratified by CKM stages, with demographic and clinical data described as actual frequency, mean ± standard deviation (SD), or median [inter-quartile range (IQR)], where appropriate. Baseline demographic and clinical data were compared between the patients with different stages of CKM syndrome using one-way ANOVA.

To compare the risk of mortality and serious in-hospital complications between patients hospitalized with different stages of CKM syndrome, we first performed univariate logistic regression analyses followed by multivariable analysis. The covariates adjusted in the multivariable analyses included age (as continuous variable); sex; history of malignancy; underlying chronic airway diseases (asthma, COPD, bronchiectasis); any influenza vaccine, PPSV23 or PCV13 received; and baseline blood lymphocyte and albumin level. For multivariable analyses of acute kidney injury (AKI), exposure to nephrotoxic drugs (NSAIDs, vancomycin, and piperacillin–tazobactam and aminoglycoside) during the index admission was also adjusted.

As pneumococcal disease can be prevented through vaccination, and different pneumococcal vaccines are available, we performed subgroup analysis based on the vaccination status. Subgroup analysis was conducted among patients who had received PCV13 and/or PPSV23 within the 12 months preceding the index admission date. The vaccination record was retrieved from the immunization record in the CDARS, including the type of vaccination and date of administration.

All statistical analyses were performed using the 28th version of SPSS statistical package (IBM corp., Armonk, NY, USA). Statistical significance was assessed at an α level of 0.05. STROBE and RECORD reporting guidelines were followed in the generation of this report.

### 2.4. Ethical Considerations

This study was approved by the Institutional Review Board (IRB) of the University of Hong Kong and Hospital Authority Hong Kong West Cluster (UW 24-137). Patient informed consent was waived in this retrospective study by the IRB as it is a retrospective study without active patient recruitment, while the data were already de-identified. This study was conducted in compliance with the Declaration of Helsinki.

## 3. Results

### 3.1. Patients’ Characteristics

A total of 2192 adult patients were hospitalized for pneumococcal pneumonia to public hospitals in Hong Kong during the period of 1 January 2016 to 31 December 2024 (Table 1). There were 1005 (45.8%) patients with stage 0 to 1 CKM syndrome, 373 (17.0%) patients with stage 2 to 3 CKM syndrome, 684 (31.2%) with stage 4a CKM syndrome and 130 (5.9%) patients with stage 4b CKM syndrome.

### 3.2. Stages of CKM and Death During Index Admission

**Figure 1:**
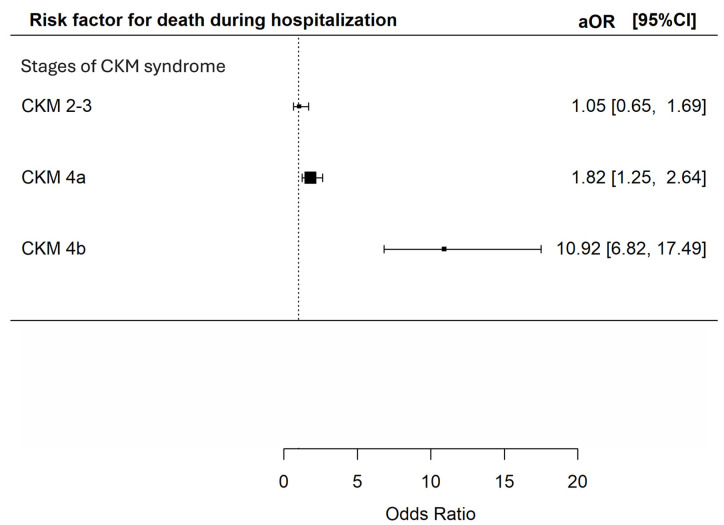
Risk of death during index admission during hospitalization among patients with different CKM syndrome stages. The *X*-axis represents the adjusted odds ratio with 95% confidence interval for death during index hospitalization of the included patients in multivariable analyses adjusted for age; sex; history of malignancy; underlying chronic airway diseases (asthma, COPD, bronchiectasis); any influenza vaccine, PPSV23 or PCV13 received; and baseline blood lymphocyte and albumin level. The *Y*-axis represents the stages of CKM syndrome of the included patients. CKM: cardiovascular–kidney–metabolic; aOR: adjusted odds ratios; CI: confidence interval.

In total, 267 (12.2%) of the patients died during index admission, with 66 (6.6%), 33 (8.8%), 114 (16.7%) and 54 (41.5%) at CKM syndrome stage 0–1, 2–3, 4a and 4b respectively. Using CKM syndrome stage 0–1 as the reference group, univariate analysis indicated that CKM syndrome stage 4a and 4b were both associated with an increased risk of death during index admission [unadjusted odds ratios (OR) 2.85 (95% CI 2.07–3.92) and 10.11 (95% CI 6.58–15.52) respectively, *p* < 0.001 for both]. The presence of CKM syndrome stage 2–3 did not appear to increase the risk of death during index admission [unadjusted OR 1.38 (95% CI 0.89–2.14, *p* = 0.15)]. Multivariable analysis further confirmed that CKM stage 4a and 4b were both independent predictors for death during index admission [adjusted OR 1.82 (95% CI 1.25–2.64) and 10.92 (95% CI 6.82–17.49) respectively (*p* = 0.002 and < 0.001)] (Figure 1).

### 3.3. Stage of CKM Syndrome and SRF

In total, 469 (21.4%) of the patients developed SRF, with 151 (15.0%), 71 (19.0%), 141 (20.6%) and 106 (81.5%) at CKM syndrome stage 0–1, 2–3, 4a and 4b respectively.

**Figure 2:**
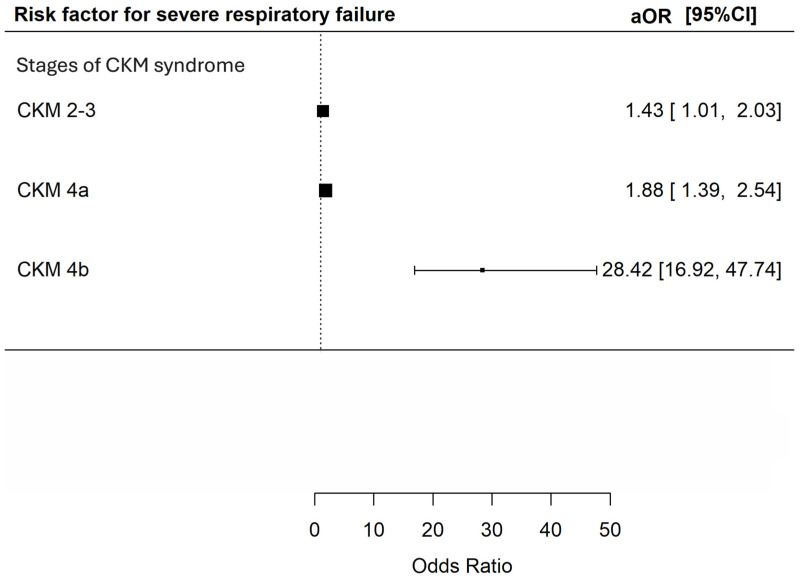
Risk of severe respiratory failure during index admission during hospitalization among patients with different CKM syndrome stages. The *X*-axis represents the adjusted odds ratio with 95% confidence interval for severe respiratory failure in index admission during hospitalization of the included patients in multivariable analyses adjusted for age; sex; history of malignancy; underlying chronic airway diseases (asthma, COPD, bronchiectasis); any influenza vaccine, PPSV23 or PCV13 received; and baseline blood lymphocyte and albumin level. The *Y*-axis represents the stages of CKM syndrome of the included patients. CKM: cardiovascular–kidney–metabolic; aOR: adjusted odds ratios; CI: confidence interval.

Univariate analysis showed that CKM stage 4a and 4b were both risk factors for SRF [OR 1.47 (95% CI 1.14–1.89) and 24.98 (95% CI = 15.52–40.19) respectively, *p* = 0.003 and <0.001]. CKM stage 2–3 did not appear to increase the risk of SRF [unadjusted OR 1.33 (95% CI 0.97–1.82, *p* = 0.073)]. However, multivariable analysis demonstrated that CKM stage 2–3, 4a and 4b were all independent predictors for SRF [adjusted OR 1.43 (95% CI 1.01–2.03), 1.88 (95% CI 1.39–2.54) and 28.42 (95% CI 16.92–47.74) respectively, *p* = 0.042, <0.001 and <0.001] (Figure 2).

### 3.4. Stages of CKM Syndrome and AKI

**Figure 3:**
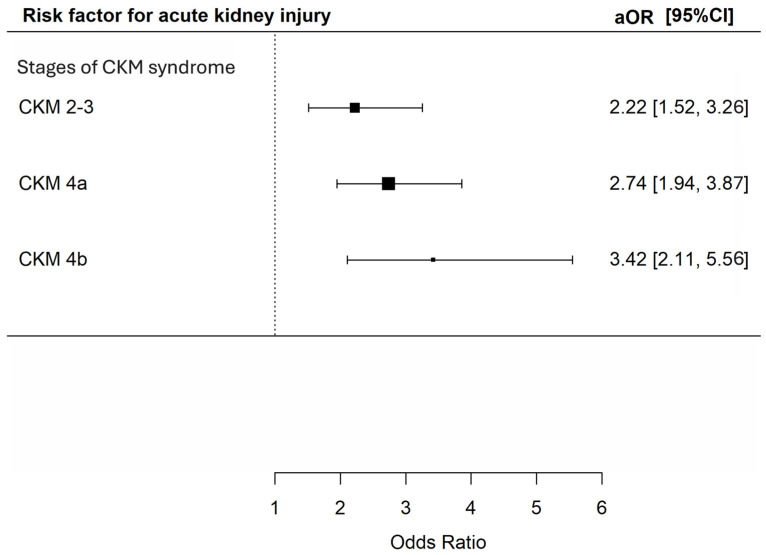
Risk of acute kidney injury during hospitalization among patients with different CKM syndrome stages. The *X*-axis represents the adjusted odds ratio with 95% confidence interval for acute kidney injury during hospitalization of the included patients in multivariable analyses adjusted for age; sex; history of malignancy; underlying chronic airway diseases (asthma, COPD, bronchiectasis); any influenza vaccine, PPSV23 or PCV13 received; baseline blood lymphocyte and albumin level and exposure to nephrotoxic drugs (NSAIDs, vancomycin, and piperacillin–tazobactam and aminoglycoside) during the index admission. The *Y*-axis represents the stages of CKM syndrome of the included patients. CKM: cardiovascular–kidney–metabolic; aOR: adjusted odds ratios; CI: confidence interval.

In total, 347 (15.8%) patients developed AKI, with 76 (7.6%), 74 (19.8%), 157 (23.0%) and 40 (30.8%) at CKM syndrome stage 0–1, 2–3, 4a and 4b respectively. There were 209 (9.5%), 291 (13.3%), 107 (4.9%), and 1160 (52.9%) patients prescribed with non-steroidal anti-inflammatory drugs, vancomycin, aminoglycoside and piperacillin/tazobactam in the cohort. Univariate analysis revealed that CKM syndrome stage 2–3, 4a and 4b were all associated with increased risk of AKI [unadjusted OR 3.03 (95% CI 2.14–4.27), 3.64 (95% CI 2.71–4.89) and 5.43 (95% CI = 3.50–8.43), *p* < 0.001 for all]. Multivariable analysis consistently showed that CKM syndrome stage 2–3, 4a and 4b were all significantly associated with increased risk of AKI [adjusted OR 2.22 (95% CI 152–3.26), 2.74 (95% CI 1.95–3.87) and 3.42 (95% CI 2.11–5.56, *p* < 0.001 for all)] (Figure 3).

### 3.5. Subgroup Analysis

Subgroup analyses were performed in patients who had or had not received pneumococcal vaccines, which consisted of 282 and 1910, patients respectively. The results in both subgroups were largely consistent with the primary analysis.

Among patients who received either PCV or PPSV, patients who had stage 4b CKM syndrome had significantly increased risks of death during hospitalization, SRF and AKI; while patients with stage 4a CKM syndrome had a significantly increased risk of death during hospitalization (Supplementary Table S1).

Among patients who had not received PCV or PPSV, patients who had stage 4a and 4b CKM syndrome had significantly increased risks of death during hospitalization, SRF and AKI, while patients with stage 2–3 CKM syndrome had a significantly increased risk of AKI (Supplementary Table S2).

## 4. Discussion

Our study demonstrated that CKM syndrome, a new entity stratifying the cardio–renal–metabolic risk profile of the patients, is an independent risk factor for severe in-hospital outcomes among patients hospitalized for pneumococcal pneumonia. We observed that a higher stage of CKM syndrome was associated with increased risks of severe in-hospital outcomes, including death during the index admission, SRF and AKI. The adverse effects of CKM were consistent in patients who had or had not received pneumococcal vaccines, highlighting the importance of optimizing CKM conditions to prevent adverse outcomes of pneumococcal infections.

The findings of this study provide possible insights into the care of patients with CKM syndrome. A higher CKM syndrome stage reflects the presence of more cardio–renal–metabolic co-morbidities or more severe co-morbidities. Optimizing the control of CKM syndrome could improve cardio–renal–metabolic health. Whether it also confers benefits in terms of respiratory health or infection outcomes remains to be determined, given the negative outcomes as reported in other body systems. Among patients with CKM syndrome, regular monitoring of relevant parameters and organ function, as well as lifestyle modification with weight control, are simple measures that should be adopted. For patients with established CKM syndrome, timely and appropriate medical therapy does not need to be overemphasized. Uncontrolled co-morbidities in CKM syndrome could result in hyperglycemia, and renal/cardiac function decline, which may exaggerate the outcomes upon contracting infections such as pneumococcal pneumonia.

The association between CKM syndrome and severe in-hospital outcomes in pneumococcal pneumonia could be mediated by diabetes mellitus and chronic kidney disease (CKD), which are known to be associated with impaired host immunity [21,22]. Diabetes mellitus is a chronic, mildly inflammatory state, while the high-glucose environment in diabetic patients can lead to immune dysfunction, making them vulnerable to infections [23,24]. Adverse outcomes among diabetic patients with infections were also reported, partly due to disturbance in innate immunity [25,26]. In patients with CKD, the immune system is also impaired, with protein-bound uremic toxins being one of the contributors [27]. At the same time, cardiovascular diseases have also been well reported to be risk factors for severe respiratory tract infections [28]. While individual components within CKM syndrome can confer risks in pneumococcal pneumonia, the combination of these factors may act synergistically, leading to elevated risks for severe in-hospital outcomes associated with pneumococcal pneumonia. Using CKM syndrome stages can help clinicians stratify the risks of patients hospitalized for pneumococcal pneumonia. By simply counting the number of conditions within CKM syndrome, clinicians can easily stratify the patients into different risk groups and estimate their risks of severe systemic and respiratory outcomes.

Various medical therapies are recommended at different CKM stages for different disease entities [29], including aspirin, statin, mineralocorticoid receptor antagonist, angiotensin-converting enzyme inhibitor/angiotensin II receptor blocker, sodium/glucose cotransporter-2 (SGLT2) inhibitors and glucagon-like peptide-1 (GLP-1) agonists. Finally, adequate control for blood pressure and diabetes mellitus is an important measure for patients at different stages of CKM syndrome. Whether the treatment for CKM syndrome could also benefit patients who have pneumococcal pneumonia requires further evaluation.

In this study, we noted that the pneumococcal vaccine uptake rate was low, which was also reported by another local study [30]. The importance of pneumococcal vaccination among elderly patients, especially those with co-morbidities, cannot be overstated. It is crucial to encourage patients with CKM syndrome to complete pneumococcal vaccination, given the fact that they are at risk of several in-hospital outcomes associated with pneumococcal pneumonia. Another interesting phenomenon observed was that despite pneumococcal vaccination, patients with CKM syndrome still developed severe in-hospital outcomes. The postulated reasons include incomplete protection with either PCV or PPSV [30,31], as well as the emergence of non-vaccine covered serotypes that can cause severe infections [31]. With the development of newer pneumococcal vaccines, such as PCV15, PCV20 and PCV21, the latter problem may at least be able to be partially resolved. Nonetheless, pneumococcal vaccination should be reinforced for patients with CKM syndrome given the high rate of severe in-hospital outcomes among these patients.

There are several limitations in this study that need to be addressed. Firstly, disease severity scores such as the Sequential Organ Failure Assessment (SOFA) Score or APACHE were not available. Nevertheless, our multivariable analysis adjusted for medical co-morbidities and disease severity. We have also performed subgroup analyses based on pneumococcal vaccination status to ensure our data are robust across different subgroups. Furthermore, our data were derived from a territory-wide electronic health record system that captures comprehensive clinical information of all adults hospitalized for pneumococcal pneumonia during the study period and are therefore a good representation of real-world data of this clinical entity.

## 5. Conclusions

CKM syndrome, especially at a higher stage, was an independent risk factor for severe in-hospital outcomes among adults hospitalized for pneumococcal pneumonia, including in-patient mortality, SRF and AKI. Subgroup analysis showed consistent results among those who have or have not received a pneumococcal vaccine within the 12 months preceding the index admission date. The results call for adequate protection of patients with CKM syndrome, especially those at a higher stage, against pneumococcal pneumonia.

## Figures and Tables

**Table 1:** Baseline clinical characteristics for the full cohort.

	All	By CKM Syndrome Stages				*p*-Value
		0–1	2–3	4a	4b	
Number of subjects (N)	2192	1005	373	684	130	
Age, years (Mean ± SD)	69.5 ± 16.9	61.6 ± 17.8	74.1 ± 13.1	78.8 ± 11.8	68.2 ± 11.6	<0.001 *
Male (N, (%))	1422 (64.9%)	656 (65.3%)	226 (60.6%)	443 (64.8%)	97 (74.6%)	0.037 *
Body mass index, kg/m^2^ (Mean ± SD)	23.1 ± 4.5	22.1 ± 4.0	23.4 ± 4.4	23.5 ± 4.6	24.8 ± 5.5	<0.001 *
Hypertension (N, (%))	705 (32.2%)	0(0%)	279 (74.8%)	376 (55.0%)	50 (38.5%)	<0.001 *
Hyperlipidemia (N, (%))	267 (12.2%)	0(0%)	90 (24.1%)	152 (22.2%)	25 (19.2%)	<0.001 *
Atrial fibrillation (N, (%))	261 (11.9%)	0(0%)	0(0%)	236 (34.5%)	25 (19.2%)	<0.001 *
Ischemic heart disease (N, (%))	261 (11.9%)	0(0%)	0(0%)	237 (34.6%)	24 (18.5%)	<0.001 *
Heart failure (N, (%))	174 (7.9%)	0(0%)	0(0%)	162 (23.7%)	12 (9.2%)	<0.001 *
History of stroke (N, (%))	331 (15.1%)	0(0%)	0 (0%)	315 (46.1%)	16 (12.3%)	<0.001 *
Diabetes mellitus (N, (%))	432 (19.7%)	0(0%)	182 (48.8%)	212 (31.0%)	38 (29.2%)	<0.001 *
Asthma (N, (%))	105 (4.8%)	34 (3.4%)	22 (5.9%)	44 (6.4%)	5 (3.8%)	0.022 *
Chronic obstructive pulmonary disease (N, (%))	281 (12.8%)	93 (9.3%)	59 (15.8%)	118 (17.3%)	11 (8.5%)	<0.001 *
Bronchiectasis (N, (%))	92 (4.2%)	44 (4.4%)	20 (5.4%)	22 (3.2%)	6 (4.6%)	0.39
Malignancies (N, (%))	254 (11.6%)	109 (10.8%)	51 (13.7%)	81 (11.8%)	13 (10.0%)	0.48
Influenza vaccine (N, (%))	686 (31.3%)	232 (23.1%)	159 (42.6%)	250 (36.5%)	45 (34.6%)	<0.001 *
Pneumococcal conjugate vaccines (N, (%))	216 (9.9%)	80 (8.0%)	54 (14.5%)	70 (10.2%)	12 (9.2%)	0.004 *
Pneumococcal polysaccharide vaccine (N, (%))	103 (4.7%)	38 (3.8%)	27 (7.2%)	34 (5.0%)	4 (3.1%)	0.043 *
Serum creatinine, µmol/L (Mean ± SD)	89.0 ± 68.6	75.8 ± 22.7	89.8 ± 70.0	88.8 ± 40.6	172.0 ± 205	<0.001 *
Serum albumin, g/L (Mean ± SD)	34.5 ± 6.8	33.8 ± 7.1	34.9 ± 6.6	35.8 ± 6.1	31.8 ± 7.5	<0.001 *
Blood leucocyte count, 10^9^/L (Mean ± SD)	18.8 ± 9.0	18.2 ± 8.4	17.8 ± 8.3	18.9 ± 8.7	24.9 ± 13.1	<0.001 *
Blood neutrophil count, 10^9^/L (Mean ± SD)	16.2 ± 8.4	15.8 ± 8.2	15.4 ± 7.6	16.4 ± 8.4	21.3 ± 10.8	<0.001 *
Blood lymphocyte count, 10^9^/L (Mean ± SD)	2.0 ± 1.7	1.9 ±1.3	1.9 ± 1.0	2.0 ± 1.8	2.4 ± 2.2	0.003 *
Blood CRP level, mg/dL (Mean ± SD)	17.2 ± 13.5	17.8 ± 13.4	18.3 ± 13.4	15.2 ± 12.7	20.5 ± 15.5	<0.001 *

Footnote: CKM, cardiovascular–kidney–metabolic; SD, standard deviation; * statistically significant; µmol, micromole; g, grams; L, liters; CRP, C-reactive protein.

## Data Availability

All available data are presented in the manuscript, and no additional data will be provided.

## Supplementary Materials

The following supporting information can be downloaded at: https://www.mdpi.com/article/10.3390/microorganisms14020439/s1, Supplementary Table S1: Severe in-hospital outcome patients who have received either PCV or PPSV; Supplementary Table S2: Severe in-hospital outcome patients who have not received PCV or PPSV.

## Abbreviations

The following abbreviations are used in this manuscript:

CKM	Cardiovascular–Kidney–Metabolic
AKI	Acute Kidney Injury
SRF	Severe Respiratory Failure
CDARS	Clinical Data Analysis and Reporting System
HA	Hospital Authority
COPD	Chronic Obstructive Pulmonary Disease
aOR	adjusted Odds Ratio
AHA	American Heart Association
CKD	Chronic Kidney Disease
SGCT2	Sodium/Glucose Cotransporter-2
GLP1	Glucagon-Like Peptide-1
SOFA	Sequential Organ Failure Assessment
APACHE	Acute Physiology and Chronic Health Evaluation
