# Supplementary material for: Cardiovascular–Kidney–Metabolic (CKM) Syndrome as Independent Risk Factor for Pneumococcal Pneumonia: Evidence from a Territory-Wide Study
Source: Microorganisms. 2026 Feb 12;14(2):439. doi: 10.3390/microorganisms14020439 (PMC12943617; doi:10.3390/microorganisms14020439)
Supplement: Supplementary file 1 [file microorganisms-14-00439-s001.zip › microorganisms-4122261-supplementary.pdf]

Supplementary Table S1. Severe in-hospital outcome patients who have received either PCV or PPSV.

Outcomes	Patient groups	Number of subjects	Univariate analysis			Multivariable† analysis		
			OR	95 % CI	p-value	aOR	95 % CI	p-value
**Death during hospitalization***	**Stage 0–1 CKM syndrome (n = 104)**	1 (1.0%)	-Reference-			-Reference-		
	**Stage 2-3 CKM syndrome (n = 70)**	2 (2.9%)	1.18	1.02–1.35	<0.001	2.17	0.17–27.19	0.55
	**Stage 4a CKM syndrome (n = 93)**	15 (16.1%)	2.18	1.91–2.49	0.37	12.31	1.33–114.38	0.02
	**Stage 4b CKM syndrome (n = 15)**	4 (26.7%)	4.35	3.48–5.43	0.003	65.68	5.87–734.96	<.0.001
**Severe respiratory failure***	**Stage 0–1 CKM syndrome (n = 104)**	26 (15.0%)	-Reference-			-Reference-		
	**Stage 2-3 CKM syndrome (n = 70)**	11 (15.7%)	1.28	1.15–1.42	< 0.001	0.86	0.36–2.01	0.72
	**Stage 4a CKM syndrome (n = 93)**	23 (24.7%)	3.02	2.73–3.35	0.26	1.60	0.73–3.48	0.24
	**Stage 4b CKM syndrome (n = 15)**	13 (86.7%)	9.11	7.69–10.79	< 0.001	27.59	5.52–138.06	<.0.001

**AKI***	**Stage 0–1 CKM syndrome (n = 104)**	17 (16.3%)	-Reference-			-Reference-		
	**Stage 2-3 CKM syndrome (n = 70)**	12 (17.1%)	1.46	1.37–1.56	0.09	1.48	0.56–3.94	0.43
	**Stage 4a CKM syndrome (n = 93)**	22 (23.7%)	4.23	3.96–4.52	< 0.001	2.37	0.94–5.96	0.07
	**Stage 4b CKM syndrome (n = 15)**	7 (46.7%)	5.69	4.86–6.66	< 0.001	4.03	1.07–15.21	0.04

Supplementary Table S2. Severe in-hospital outcome patients who have not received PCV or PPSV.

Outcomes	Patient groups	Number of subjects	Univariate analysis			Multivariable† analysis		
			OR	95 % CI	p-value	aOR	95 % CI	p-value
**Death during hospitalization***	**Stage 0–1 CKM syndrome (n = 901)**	65 (7.2%)	-Reference-			-Reference-		
	**Stage 2-3 CKM syndrome (n = 303)**	31 (10.2%)	1.47	0.94–2.30	0.095	1.06	0.65–1.74	0.82
	**Stage 4a CKM syndrome (n = 591)**	99 (16.8%)	2.59	1.86–3.61	<0.001	1.55	1.05–2.28	0.028
	**Stage 4b CKM syndrome (n = 115)**	50 (43.5%)	9.89	6.33–15.47	<0.001	10.19	6.23–16.64	<0.001
**Severe respiratory failure***	**Stage 0–1 CKM syndrome (n = 901)**	125 (13.9%)	-Reference-					
	**Stage 2-3 CKM syndrome (n = 303)**	60 (19.8%)	1.53	1.09–2.15	0.014	1.39	0.98–1.99	0.68
	**Stage 4a CKM**	118 (20.0%)	1.55	1.18–2.04	0.002	1.57	1.18–2.08	0.002

	**syndrome (n = 591)**							
	**Stage 4b CKM syndrome (n = 115)**	93 (80.9%)	26.24	15.89–43.34	< 0.001	28.62	16.83–48.68	<0.001
**AKI***	**Stage 0–1 CKM syndrome (n = 901)**	59 (6.5%)	-Reference-					
	**Stage 2-3 CKM syndrome (n = 303)**	62 (20.5%)	3.67	2.50–5.39	< 0.001	2.59	1.69– 3.96	<0.001
	**Stage 4a CKM syndrome (n = 591)**	135 (22.8%)	4.23	3.05–5.86	< 0.001	3.03	2.07–4.431	<0.001
	**Stage 4b CKM syndrome (n = 115)**	33 (28.7%)	5.74	3.55–9.31	< 0.001	3.42	2.00–5.84	<0.001
